# Near-field optical trapping in a non-conservative force field

**DOI:** 10.1038/s41598-018-36653-0

**Published:** 2019-01-24

**Authors:** Mohammad Asif Zaman, Punnag Padhy, Lambertus Hesselink

**Affiliations:** Department of Electrical Engineering, Stanford University, Stanford, CA 94305 USA

## Abstract

The force-field generated by a near-field optical trap is analyzed. A C-shaped engraving on a gold film is considered as the trap. By separating out the conservative component and the solenoidal component of the force-field using Helmholtz-Hodge decomposition, it was found that the force is non-conservative. Conventional method of calculating the optical potential from the force-field is shown to be inaccurate when the trapping force is not purely conservative. An alternative method is presented to accurately estimate the potential. The positional statistics of a trapped nanoparticle in this non-conservative field is calculated. A model is proposed that relates the position distribution to the conservative component of the force. The model is found to be consistent with numerical and experimental results. In order to show the generality of the approach, the same analysis is repeated for a plasmonic trap consisting of a gold nanopillar. Similar consistency is observed for this structure as well.

## Introduction

Trapping of micron and submicron sized particles have received significant attention in several branches of science including biotechnology^1–3^, physics^4^, and chemistry^5^. Optical trapping and manipulation schemes are playing an important role in the operation of lab-on-a-chip (LOC) devices^6^. Accurate modeling of optical trapping forces are becoming more and more important for the design process as the complexity of such devices increase.

Free space optical trapping can be achieved by tightly focusing a laser beam. Such setups are known as *optical tweezers* where particles become trapped near the focus point of the laser beam due to the gradient forces^7–9^. To apply sufficient gradient force for successful trapping, the spot size of the focused laser beam must be comparable to the size the particle. As a result, the smallest particle that can be trapped is determined by the diffraction limit. To overcome this limitation, near-field optical traps have been developed. Such traps use the evanescent fields generated by plasmonic nanostructures^10–12^ or dielectric waveguides^2^ to create the gradient forces. Unlike propagating fields, evanescent fields can be focused beyond the diffraction limit^11^, making it possible to trap particles smaller than the limit of optical tweezers. Moreover, compared to the three-dimensional nature of optical tweezers, near-field traps, such as plasmonic traps, are planar in nature. This makes it possible to fabricate multiple plasmonic traps on a single substrate for LOC applications^13^. By using multiple plasmonic traps on a chip, it is possible to achieve controlled manipulation of nanoparticles^14,15^. Designing such a complex system requires fast and accurate characterization of the force-field and the particle motion near a plasmonic trap.

In this paper, we will investigate the nature of the force-field generated by a plasmonic trap and how it effects the trapping dynamics of a dielectric nanoparticle. We will mainly focuse on a plasmonic trap consisting of a C-shaped engraving (CSE) on a gold film. C-shaped structures produce strong localized field intensity enhancement and can focus light very tightly (<*λ*/10)^16,17^. This can create a strong gradient force that can be utilized for particle trapping. As the CSE geometry is asymmetric, the excitation of the structure can be controlled by changing the polarization of the incident light. Due to these favorable characteristics, CSEs have been successfully used for plasmonic trapping and manipulation schemes^14,15^. To show that the findings presented in this paper is not limited CSE only, we also consider a second plasmonic structure consisting of a cylindrical gold nanopillar. This is also a well known structure that has been successfully used for near-field trapping^10,18^.

We have computed the force a dielectric nanoparticle experiences near a CSE and near a nanopillar. While analyzing this force-field, we have uncovered some interesting properties of plasmonic traps that were not prevously reported in the literature. The force-field is decomposed into a conservative/irrotational component and a non-conservative/solenoidal component using the Helmholtz-Hodge decomposition (HHD). The force-field is found to have non-negligible solenoidal component and thus cannot be considered as a purely conservative field. This finding is contrary to the commonly used assumption of the optical trapping force-field to be conservative^19,20^. This assumption directly affects how the optical *trapping potential* is calculated, which is often used to characterize optical traps. For a conservative field, this trapping potential can be calculated using a line integral. For fields with significant solenoidal component, such an approach may not be accurate. We show that for the plasmonic traps under consideration, this approach of calculating the trapping potential can give significantly erroneous results. It should be mentioned that direct line integration has been used to calculate the trapping potential of near-field traps in many published literature without verifying whether the force-field is conservative or not^10,19–21^. Since the two plasmonic structures we considered here both generate solenoidal force components, it is likely that other structures may also exhibit similar characteristics. In this paper, we present an HHD based method of calculating the trapping potential. It is found that the potential obtained from the proposed approach represents the trap characteristics more accurately. Since trap stiffness, trapping range and many other characterizing parameters of an optical trap are often extracted from the trapping potential, accurate estimation of the trapping potential is of significant importance.

In addition to proposing a method of estimating the trapping potential more accurately, we also relate the position distribution of a trapped nanoparticle with the trapping potential. It is well known that the position of a particle trapped in a potential well created by a conservative force-field follows a Boltzmann distribution^9^. No similar relationship has been proposed for non-conservative force-fields. We posit that for a trapping force-field which is not purely conservative (but the conservative component dominates the solenoidal component), the Boltzmann function can still be used to model the position distribution. If the trapping potential is obtained from the HHD and is used as the argument of the Boltzmann distribution, it can accurately model the position of a trapped nano-particle. We present numerical and experimental data that support our claims. Only a few works have investigated whether a trapping force-field is conservative or not^22–24^. However, these works focused mostly on optical tweezers. To the best of our knowledge, our group is the first to apply the HHD method to analyze near-field traps^25^. A detailed analysis on how the trapping potential for a non-conservative force-field can be calculated and used to characterize the position distribution of a trapped nanoparticle has not appeared in the literature. By calculating the position distribution using the proposed method, it is possible to estimate the trapping range (area of influence)^26^ of a plasmonic trap. This can be very useful in the design process of LOC systems containing multiple traps as the necessary spacing between the traps can be estimated from the trapping range.

The paper is organized as follows. First, the geometry of the plasmonic traps are described. Then the optical simulation and force calculations are discussed. It is followed by the formulation of the motion of a nanoparticle near the trap. After that, the decomposition of the force-field and the Boltzmann model are discussed. The experimental setup is described next. And finally, the results discussed.

### Geometry of the structures

**Figure 1:**
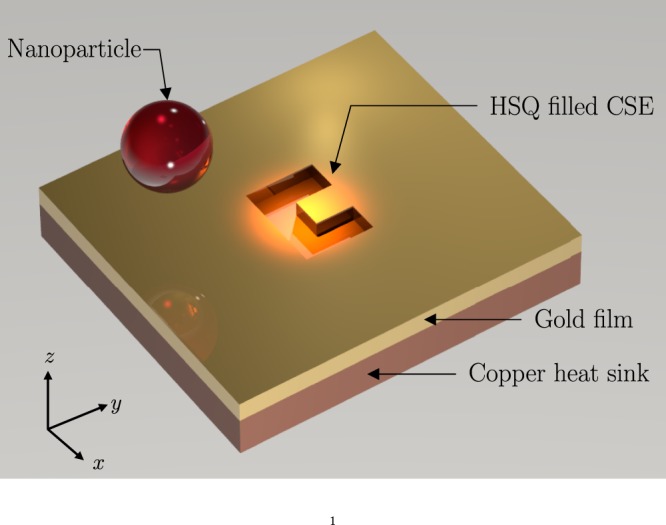
Near-field trapping using a C-shaped engraving.

**Figure 2:**
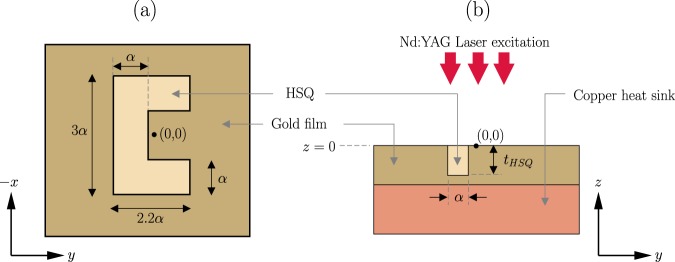
Geometry of the C-shaped engraving.

**Figure 3:**
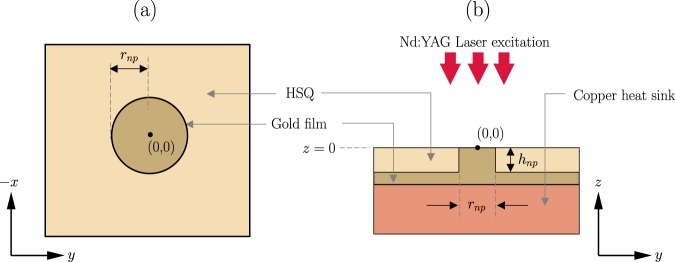
Geometry of the gold nanopillar.

A three-dimensional representation of the main plasmonic trap under consideration is shown in Fig. 1. The first plasmonic trap consists of a CSE on a gold film. The engraving is filled with Hydrogen silsesquioxane (HSQ). The structure is illuminated from the top by a focused 1064 nm Nd:YAG laser. A copper heat sink is placed below the gold film to reduce thermal effects^10,15^. The top and cross-sectional view of the structure along with the coordinate system used, are shown in Fig. 2. A second plasmonic trap consisting of a cylindrical gold nanopillar was also considered. The geometry of that structure is shown in Fig. 3. The illumination method for the nanopillar is identical to that of the CSE.

Colloidal solution of nanoparticles in water medium is placed on top of the structure. Fluorescent polystyrene spheres of radius 150 nm are used as the nanoparticles. This is a common choice for many biological applications^27,28^. The geometry of the CSE is defined by the characteristic parameter, *α* and the depth of the engraving, *t*_*HSQ*_. The nanopillar geometry is defined by its radius, *r*_*np*_ and height, *h*_*np*_. The values of the parameters are listed in Table 1. The parameter values are selected such that the structures are resonant at wavelengths near 1064 nm.

**Table 1:** Geometrical and Material parameters.

Parameter	Value
CSE characteristic parameter, *α*	60 nm
Depth of the engraving, *t*_*HSQ*_	150 nm
Radius of the nanopillar, *r*_*np*_	150 nm
Height of the nanopillar, *h*_*np*_	150 nm
Radius of polystyrene nanoparticle, *r*_*o*_	150 nm
Refractive index of water, *n*_*w*_	1.33
Refractive index of HSQ, *n*_*HSQ*_	1.4
Refractive index of polystyrene, *n*_*p*_	1.58

The optical response of the trap depends on the geometry and the material properties. The refractive index of the dielectric materials are listed in Table 1. A Drude model is used to characterize the dielectric function of gold. The model is consistent with experimental data^29^ for the wavelength range under consideration (800–1200 nm).

### Force calculation

**Figure 4:**
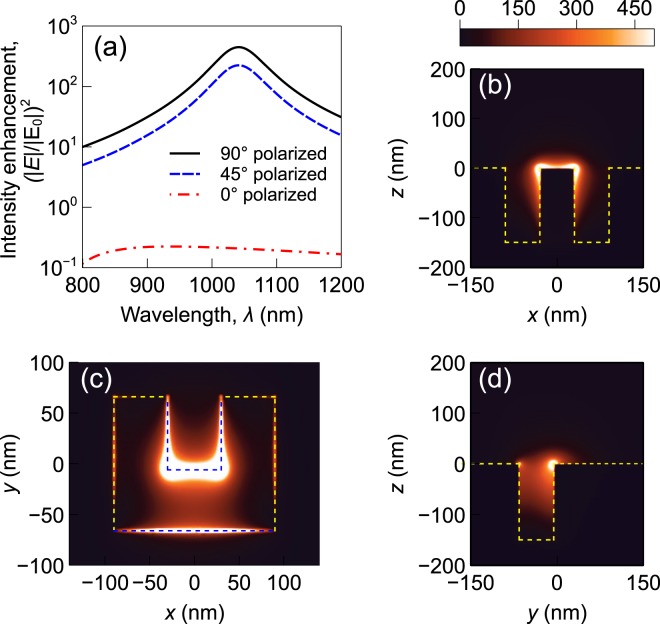
Electric field intensity enhancement near a CSE: (**a**) As a function of wavelength at the point (0,0,5 nm), (**b**) at *y* = 0 plane, (**c**) at *z* = 0 plane, and (**d**) at *x* = 0 plane. A *y*-polarized incident light of wavelength 1064 nm is considered for (**b**), (**c**) and (**d**). The plots share the same colorbar. Input intensity is set at 1 mW/*μ*m^2^.

**Figure 5:**
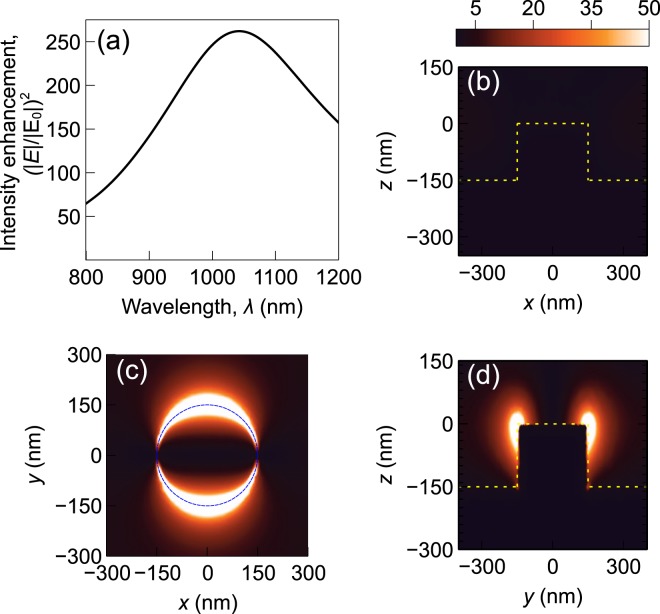
Electric field intensity enhancement near a gold nanopillar: (**a**) As a function of wavelength at the point (0,150 nm,5 nm), (**b**) at *y* = 0 plane, (**c**) at *z* = 0 plane, and (**d**) at *x* = 0 plane. A *y*-polarized incident light of wavelength 1064 nm is considered for (b), (c) and (d). The plots share the same colorbar. Input intensity is set at 1 mW/*μ*m^2^.

To find the trapping force generated by the CSE and the nanopillar, the electromagnetic field distribution near the structures must be calculated. The force depends on the gradient of the square of the electric field. A commercial finite element solver (Comsol Multiphysics) is used to simulate the optical response of the structure. The field distribution near a plasmonic structure depends on the geometry of the structure, material properties, and the wavelength and polarization of the incident light. The electric field intensity enhancement as a function of incident light wavelength is shown in Figs 4(a) and 5(a). No nanoparticle is assumed to be present in the system for this simulation. It can be observed that the intensity enhancement is maximum at wavelengths near 1064 nm and when the light is *y*-polarized (90 degree polarized). The input intensity is assumed to be 1 mW/*μ*m^2^. The wavelength, polarization and intensity of the incident light are set at these values for the rest of the paper. The intensity enhancement at different cut-planes for 1064 nm *y*-polarized light are also shown in Figs 4 and 5. It can be noted that strong localized enhancement is achieved near the center of the CSE and along the top perimeter of the nanopillar. This is consistent with published results available in the literature^10,15,18^.

To find the optical force on a nanoparticle near the CSE, the nanoparticle must be taken into account when calculating the electromagnetic field distribution. The Maxwell stress tensor (MST) method can be used to calculate the force from the fields^20,30^:

1 $${\langle {\bf{F}}\rangle }_{t}={\int }_{S}{\langle \overleftrightarrow{{\bf{T}}}\rangle }_{t}\cdot \hat{{\bf{n}}}\,{\rm{d}}S,$$

2 $$\overleftrightarrow{{\bf{T}}}={\varepsilon }_{w}({\bf{EE}}-\frac{1}{2}|{\bf{E}}{|}^{2}\overleftrightarrow{{\bf{I}}})+{\mu }_{w}({\bf{HH}}-\frac{1}{2}|{\bf{H}}{|}^{2}\overleftrightarrow{{\bf{I}}}).$$

**Figure 6:**
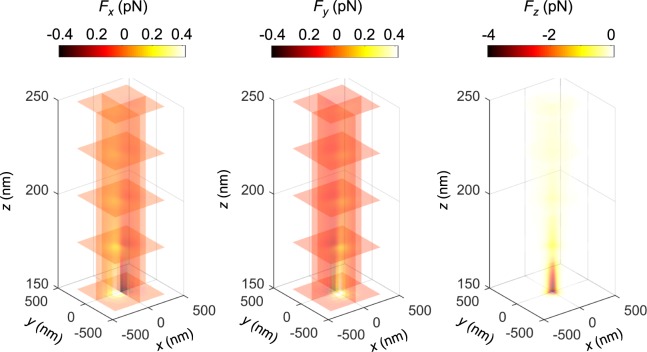
Force-field near the CSE at different slice planes.

Here, **F** is the net electromagnetic force acting on the nanoparticle, 〈⋅〉_*t*_ represents time-averaged value, *S* is the outer surface of the nanoparticle, $\hat{{\bf{n}}}$ is the surface normal to *S*, $\overleftrightarrow{{\bf{T}}}$ is the Maxwell stress tensor, **E** is electric field, **H** is the magnetic field, *ε*_*w*_ and *μ*_*w*_ are the permittivity and permeability of the surrounding medium (water), respectively, and $\overleftrightarrow{{\bf{I}}}$ is the identity tensor. For a given position of the nanoparticle, the **E** and **H** fields are calculated and Eq. 1 is used to evaluate the force at that point. The position of the nanoparticle is swept in a discrete three-dimensional grid near the plasmonic structure and the calculations are repeated to map out the force-field. Three-dimensional spline interpolation is applied to this discrete data set to evaluate the force at any arbitrary point. The calculated force near the CSE at different slice planes are shown in Fig. 6. A similar figure can be generated for the nanopillar. We have not included the figure here for brevity. The figure shows pico-newton level pulling forces towards the center of the trap at (0, 0, 0). The force generated by the nanopillar has similar magnitude and characteristics.

### Particle dynamics

Nanoparticles suspended in a liquid medium exhibit Brownian motion. Assuming a low Reynolds number environment, the motion of a nanoparticle in water under the influence of an external force-field can be modeled using the modified Langevin equation^31–33^:

3 $$\dot{{\bf{r}}}(t)=\frac{\overleftrightarrow{{\bf{D}}}({\bf{r}})}{{k}_{B}T}{\bf{F}}({\bf{r}},t)+\sqrt{2}{\overleftrightarrow{{\bf{D}}}}_{\frac{1}{2}}({\bf{r}}){\bf{W}}(t\mathrm{).}$$

Here, **r** is the position of the center of the nanoparticle, **F** is the optical trapping force acting on the nanoparticle, *k*_*B*_ is the Boltzmann constant, *T* is the temperature, $\overleftrightarrow{{\bf{D}}}$ is the diffusion tensor, and **W**(*t*) is a vector white noise term. The tensor ${\overleftrightarrow{{\bf{D}}}}_{\frac{1}{2}}$ is obtained by taking element-wise square root of $\overleftrightarrow{{\bf{D}}}$. Each Cartesian component of **W**(*t*) is a Gaussian random process with zero mean and unit variance. The hydrodynamic interaction between the nanoparticle and the solid bottom surface (*z* = 0 plane) affects the motion of the nanoparticle. These effects are included in the terms of the diffusion tensor. $\overleftrightarrow{{\bf{D}}}({\bf{r}})$ is a diagonal tensor with ${D}_{11}({\bf{r}})={D}_{22}({\bf{r}})={D}_{\parallel }({\bf{r}})$, and *D*_33_(**r**) = *D*_⊥_(**r**), where the components are defined as^34–36^:

4 $${D}_{\parallel }({\bf{r}})=\frac{{k}_{B}T}{6\pi \eta {r}_{o}}(1-\frac{9{r}_{o}}{16z}+\frac{{r}_{o}^{3}}{8{z}^{3}}-\frac{45{r}_{o}^{4}}{256{z}^{4}}-\frac{{r}_{o}^{5}}{16{z}^{5}})\,,$$

5 $${D}_{\perp }({\bf{r}})=\frac{{k}_{B}T}{6\pi \eta {r}_{o}}(\frac{6{z}^{2}+2{r}_{o}z}{6{z}^{2}+9{r}_{o}z+2{r}_{o}^{2}})\,\mathrm{.}$$

Here *η* is the dynamic viscosity of the medium (water), *r*_*o*_ is the radius of the nanoparticle, and *z* is the *z*-coordinate of the center of the nanoparticle. As the separation from the *z* = 0 surface increases, the hydrodynamic interactions between the surface and the nanoparticle decreases. So, for large *z*, *D*_∥_(**r**) and *D*_⊥_(**r**) converge to the free space diffusion coefficient of *k*_*B*_*T*/6*πηr*_*o*_.

Equation 3 is a stochastic differential equation that can be discretized to obtain the following finite difference equation^32^:

6 $${{\bf{r}}}_{k+1}={{\bf{r}}}_{k}+\frac{\overleftrightarrow{{\bf{D}}}({{\bf{r}}}_{k})}{{k}_{B}T}{\bf{F}}({{\bf{r}}}_{k},{t}_{k})+\sqrt{2{\rm{\Delta }}t}{\overleftrightarrow{{\bf{D}}}}_{\frac{1}{2}}({{\bf{r}}}_{k}){{\bf{W}}}_{k}.$$

The Euler-Maruyama method can be used to solve this finite difference equation numerically^37^. A large number of independent trajectories are solved to obtain the position distribution of a trapped nanoparticle. This distribution will be compared against the proposed Boltzmann model and the experimental results later.

### Helmholtz-Hodge decomposition (HHD) and position distribution

The Helmholtz theorem states that under reasonable regularity condition, a force-field can be decomposed into a conservative component and a non-conservative/solenoidal component:

7 $${\bf{F}}=-\nabla u+\nabla \times {\bf{A}}.$$

Here −∇*u* is the conservative component and ∇ × **A** is the solenoidal component. *u* is a scalar potential function and A is a vector potential function. For a conservative force-field, the solenoidal component is zero and the scalar potential can be obtained from direct integration, ${u}_{DI}({\bf{r}})=-{\int }_{-\infty }^{{\bf{r}}}{\bf{F}}({\bf{r}}^{\prime} )\cdot d{\bf{r}}^{\prime}$. However, for fields with solenoidal component, this approach does not work. For a sufficiently smooth ***F*** defined in a bounded domain Ω with a smooth boundary ∂Ω, the HHD can be applied to separate out the components. The function *u* can be calculated by solving the following equations:

8 $$-{\nabla }^{2}u=\nabla \cdot {\bf{F}}\,{\rm{on}}\,{\rm{\Omega }},$$

9 $$\nabla u\cdot \hat{{\bf{n}}}={\bf{F}}\cdot \hat{{\bf{n}}}\,{\rm{on}}\,\partial {\rm{\Omega }}.$$

**Figure 7:**
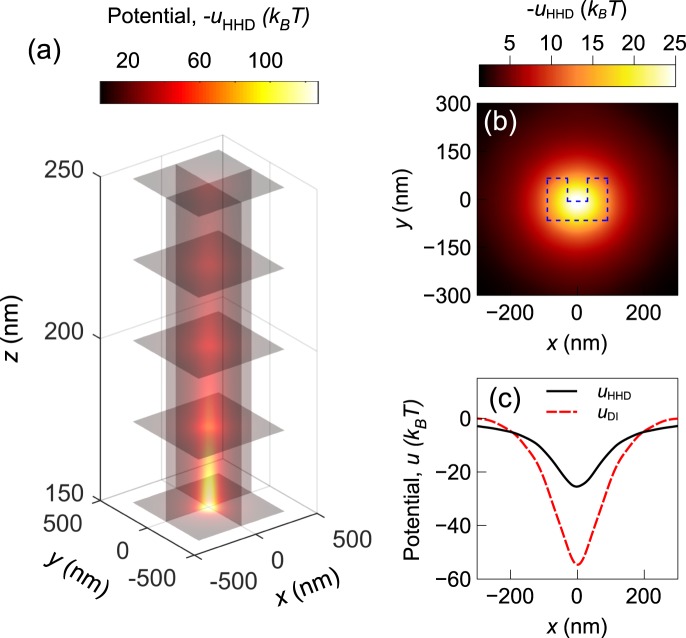
Scalar potential profile obtained from HHD for the CSE structure: (**a**) at different slice planes, (**b**) at *z* = 155 nm plane, and (**c**) along *y* = 0, *z* = 155 nm line. The potential obtained from direct integration is also shown in (**c**).

**Figure 8:**
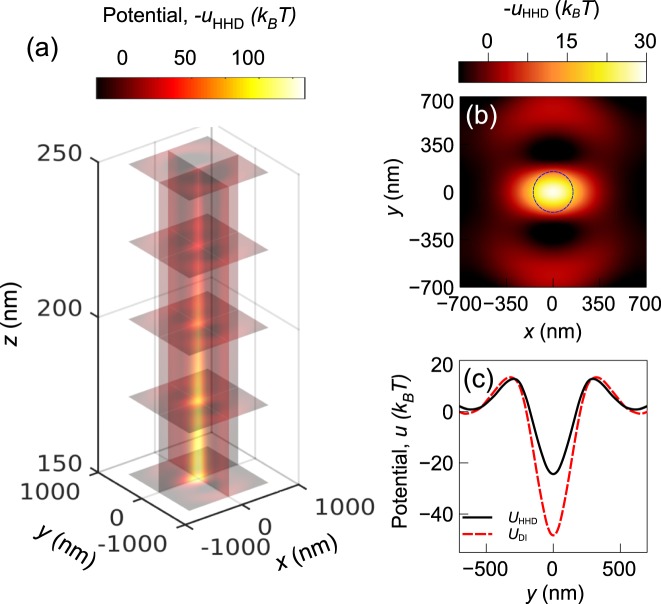
Scalar potential profile obtained from HHD for the nanopillar structure: (**a**) at different slice planes, (**b**) at *z* = 155 nm plane, and (**c**) along *x* = 0, *z* = 155 nm line. The potential obtained from direct integration is also shown in (**c**).

This is a partial differential equation with Neumann boundary conditions that can be numerically solved. The results are shown in Figs 7 and 8. From *u*, the two components: ∇*u* and ∇ × **A** can be calculated using Eq. 7. From this analysis, we have found that the force-field of a CSE has non-negligible solenoidal component. The norm of the solenoidal component is 40% of the norm of the conservative component for the CSE. For the nanopillar structure, the ratio has lower value of 24%. This finding is significant as some works have considered the force from plasmonic traps to be conservative^19–21^. Due to the solenoidal component, the scalar potential obtained from direct integration differs significantly from the one obtained from HHD. Figures 7(c) and 8(c) show the comparison between the two methods.

It can be noted that the direct integration method overestimates the potential depth by more than a factor of two. Thus, any subsequent computations based on these results may also differ significantly. The difference in the results from the two methods is larger for the CSE structure. This is expected as the solenoidal component was found to be weaker in the nanopillar structure. Our analysis shows that the *u* obtained from the HHD can be considered as a the optical trapping potential even if the force is not purely conservative.

The position distribution or the spatial extent of a trapped nanoparticle, *f*(**r**), depends on the potential function. For a conservative force-field, the Boltzmann distribution can be used to model the phenomenon^9,38–40^:

10 $$f({\bf{r}})={A}_{N}{e}^{-\frac{u({\bf{r}})}{{k}_{B}T}}.$$

Here *A*_*N*_ is a normalizing factor given by ${A}_{N}=\mathrm{1/}{\int }_{-\infty }^{\infty }{e}^{-\frac{u({\bf{r}}^{\prime} )}{{k}_{B}T}}d{\bf{r}}^{\prime}$. For a non-conservative force-field, the Boltzmann distribution is not expected to be valid. However, we have found that even though the force-field from the plasmonic trap is non-conservative, if the scalar potential obtained from the HHD is used as the argument of the Boltzmann function, the resulting distribution is consistent with numerical and experimental results. We discuss this further in the following sections.

### Experiment

**Figure 9:**
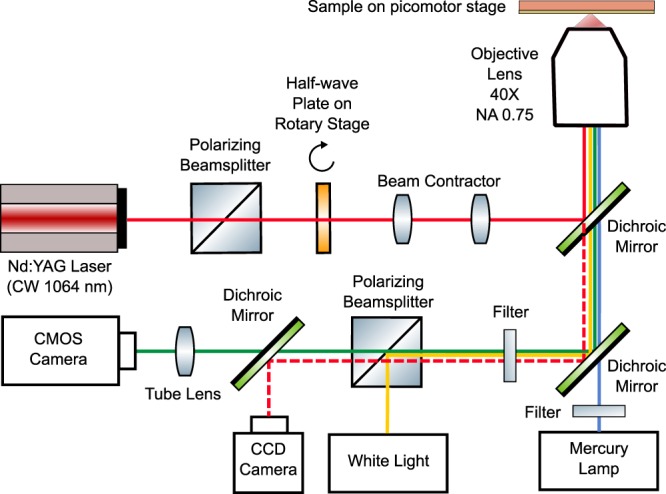
Experimental setup.

We have performed experiments to study the motion of a nanoparticle trapped on a CSE. The CSE was fabricated using the same methods as described in^15^. The experimental setup is shown in Fig. 9. A half-wave plate on a rotary stage is used to control the polarization of the laser light hitting the CSE. Fluorescence imaging is performed using an inverted microscope (Nikon Eclipse TE2000-U). A mercury lamp is used to excite the fluorescence of the polystyrene nanoparticle. A CCD camera is used to monitor the focused laser beam. A CMOS camera is used to capture video of the motion of the nanoparticle. The captured video is digitally processed for noise reduction and a particle tracking algorithm is used to calculate the position of the nanoparticle center.

The sample is prepared by placing a small droplet of dilute solution of fluorescent polystyrene beads on a cover slip. The fabricated device is placed on top of the cover slip (gold side facing down). The sample is placed on a picomotor stage. The laser power is adjusted to 1 mW/*μ*m^2^ and the half-wave plate is rotated to match the orientation of the CSE. Once a trapping event is observed, the video is recorded for further processing.

## Results and Discussion

**Figure 10:**
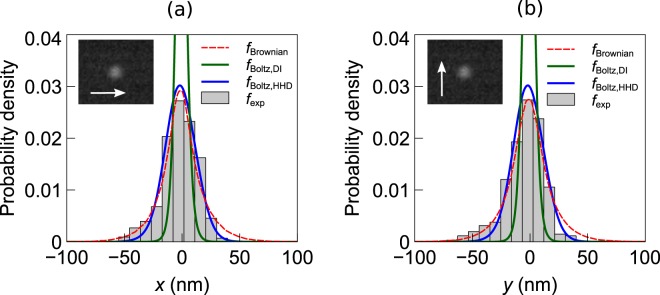
Position distribution of a trapped nanoparticle at *z* = 155 nm plane for the CSE structure: (**a**) along *x*-direction, (**b**) along *y*-direction. The inset shows a frame of the captured video. The arrows in the inset indicate the direction along which the distribution is taken.

**Figure 11:**
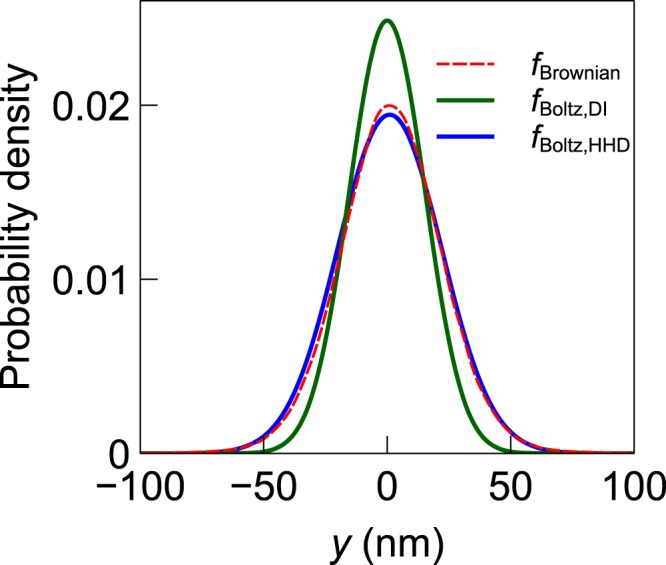
Position distribution of a trapped nanoparticle at *z* = 155 nm plane along *y*-direction for the nanopillar structure.

For the CSE structure, the position distribution of a trapped nanoparticle obtained from the experiment (*f*_exp_) is shown in Fig. 10. The numerical results from the Brownian dynamics (*f*_Brownian_) and the Boltzmann models are also shown on the same plots. The Boltzmann distribution with potential function obtained from the HHD (*f*_Boltz,HHD_) matches closely with numerical and experimental results. However, when the potential obtained from direct integration is used in the Boltzmann model (*f*_Boltz,DI_), it gives a result which differs significantly from the rest. It can be noted that the experimental distribution along *x* and *y* axis both show a slight asymmetry. Imperfections in the fabrication process may lead to such variations. The same plots for the nanopillar structure are shown in Fig. 11. Only numerical data are presented for this structure.

The results show that the potential obtained from direct integration is not a good indicator of the trap characteristics when the force-field has large solenoidal component. This is a significant finding as direct line integration is a commonly used method to estimate the optical potential^10,19,21^. As we have shown that the the trapping force-field of a CSE and a nanopillar is not purely conservative, it is reasonable to assume that other plasmonic structures may share similar properties. An analysis is necessary to check if a solenoidal component exists in the force-field. In such a case, the conservative component can be extracted from the force-field using the HHD. We have shown that the scalar potential obtained from the HHD is more representative of the trap behavior.

It should be noted that the error level in the position distribution obtained from direct integration method is smaller for the nanopillar structure than the CSE structure. Due to the smaller magnitude of the solenoidal component for the nanopillar structure, the direct integration method introduces less error in estimating the trapping potential. If the solenoidal component was zero, then the direct integration method and the HHD method would give identical results. For conservative force-fields (e.g. optical tweezers), the solenoidal component is negligible and the direct integration method can give results that are consistent with experimental values. The asymmetrical geometry of the CSE may play a role in creating larger solenoidal forces. Asymmetric geometries can create orbital angular momentum^41^ and rotational forces^42^ which indicate the presence of solenoidal forces. Even though the nanopillar structure is symmetric, the field distribution it produces is not symmetric. This is because the localized field intensity enhancement of the structure is aligned along the polarization axis of the incident light (as can be seen in Fig. 5(d)). This asymmetry in field may lead to the creation of a solenoidal component in the force profile.

Another finding is that despite the presence of a solenoidal component in the force, the Boltzmann distribution can be used to model the position distribution of a trapped nanoparticle. The model is accurate when the potential function from the HHD is used as its argument. This finding is interesting as it suggests that the steady state position distribution only depends on one of the components of the force. Despite the solenoidal component being non-negligible, only the conservative component determines the position distribution. We posit that the solenoidal component induces a rotational/spinning motion which does not affect the steady-state statistics significantly. For example, if the nanoparticle experiences some spinning motion around its axis, then the net displacement due to the spinning is expected to be close to zero (assuming no precession). The solenoidal component can be thought of as the part of the force that is not related to the net trapping force. Working under this assumption, we can concluded that the solenoidal component is unlikely to affect the particle distribution. However, further investigation is required to completely explain the phenomenon. As the conservative component solely effects the steady state particle position, it is not surprising that the distribution follows the statistics of a purely conservative field.

The position distribution/spatial spread of a nanoparticle is an important parameter for LOC system design. It determines the maximum separation between two independent traps that allows a nanoparticle to be transported from one trap to another^14,15^. To obtain position statistics from Brownian simulation, a large number of independent trajectories must be calculated. This requires large computational time. The applicability of the Boltzmann model for non-conservative force-fields opens up an alternative approach of obtaining the position distribution. The Boltzmann model requires only the computation of the potential function. Extracting the potential from the force-field using the HHD requires considerably less computational time. So, the Boltzmann model allows faster estimation of the particle position spread. This could be useful in the design process of LOC systems. If the direct integration method is used to estimate the spatial spread, it would underestimate the spread by a factor of two for the CSE structure. The corresponding LOC design would contain twice as many CSEs than actually necessary. In some cases, direct integration method may suggest a separation value smaller than what can be fabricated accurately.

Although this paper analyzes the force-field of a CSE and a nanopillar, the same analysis can be applied to other structures used for near-field trapping. The methods used for calculating the force-field and the HHD will be the same for any structure. It is reasonable to assume that other plasmonic structures may also generate non-conservative force-fields. For those cases, the results presented in this paper can be useful.

## Data Availability

The datasets generated during and/or analyzed during the current study are available from the corresponding author on reasonable request.
